# Effect of knee angle on neuromuscular assessment of plantar flexor muscles: A reliability study

**DOI:** 10.1371/journal.pone.0195220

**Published:** 2018-03-29

**Authors:** Angèle N. Merlet, Thomas Cattagni, Christophe Cornu, Marc Jubeau

**Affiliations:** 1 Laboratory Movement, Interactions, Performance, Faculty of Sport Sciences, University of Nantes, Nantes, France; 2 Inserm Unit 1179, Team 3: Technologies and Innovative Therapies Applied to Neuromuscular diseases, UVSQ. CIC 805, Physiology-Functional Testing Ward, AP-HP, Raymond Poincaré Teaching Hospital, Garches, France; University of Toronto, CANADA

## Abstract

**Introduction:**

This study aimed to determine the intra- and inter-session reliability of neuromuscular assessment of plantar flexor (PF) muscles at three knee angles.

**Methods:**

Twelve young adults were tested for three knee angles (90°, 30° and 0°) and at three time points separated by 1 hour (intra-session) and 7 days (inter-session). Electrical (H reflex, M wave) and mechanical (evoked and maximal voluntary torque, activation level) parameters were measured on the PF muscles. Intraclass correlation coefficients (ICC) and coefficients of variation were calculated to determine intra- and inter-session reliability.

**Results:**

The mechanical measurements presented excellent (ICC>0.75) intra- and inter-session reliabilities regardless of the knee angle considered. The reliability of electrical measurements was better for the 90° knee angle compared to the 0° and 30° angles.

**Conclusions:**

Changes in the knee angle may influence the reliability of neuromuscular assessments, which indicates the importance of considering the knee angle to collect consistent outcomes on the PF muscles.

## Introduction

Electrical nerve stimulation is widely used in sport medicine studies to assess the neuromuscular function [1,2]. This technique provides valuable information about the peripheral (e.g., muscle compound action potential, twitch torque) and neural (e.g., Hoffmann reflex, voluntary activation level) mechanisms that cause changes in motor/muscle activity. It is therefore important to ensure that measures of neuromuscular function are reliable so that differences observed over time may be attributable to changes in physiology, and not random variation.

By applying a stimulus on the posterior tibial nerve (in the popliteal fossa), the Hoffmann reflex (H reflex), which is a short latency electrical analogue of the monosynaptic reflex, can be recorded using electromyography (EMG) on plantar flexor muscles (PF). The H reflex is preceded by another electrophysiological response, the muscle compound action potential (M wave). It is commonly accepted that the ratio between the maximal H reflex (H_max_) and the maximal M wave (M_max_), i.e. H_max_/M_max_, represents the efficiency of spinal transmission from Ia-afferent inputs to α-motoneurons, including both excitation and inhibition activities at the spinal level [3–6]. Electrical nerve stimulation is also used to assess the voluntary activation level (VAL) by using the twitch interpolation technique and the twitch contractile properties. The twitch interpolation technique consists of applying a supramaximal electrical nerve stimulation during a maximal voluntary contraction (superimposed stimulation) [7]. If the superimposed stimulation induces a torque increase, it means that the participant is not able to fully activate its muscles [8–10]. The assessment of the twitch contractile properties consists of applying an electrical stimulation on a relaxed muscle, i.e., at rest [7].

The reliability between experimental sessions of PF neuromuscular tests has been previously investigated in the literature for some experimental approaches [11–17]. Clark et al. (2007) [11] evaluated the reliability of a large number of PF neuromuscular measurements separated by a relatively long duration of time (4 weeks). They observed high to moderate reliability for PF MVC strength, PF maximal EMG activity, VAL, M_max_ and H reflex. The reliability of neuromuscular parameters for PF over a period of two weeks was also reported by Stutzig & Siebert (2016) [17]. They revealed that a majority of the collected neuromuscular parameters (28 of 34) had moderate (ICC, 0.61–0.80) and substantial (ICC, 0.81–1.00) reliability, which led to the conclusion that these measurements are sufficiently consistent to be used in interventional studies. Clark et al. (2007) [11] and Stutzig & Siebert (2016) [17] collected their measurements at a fixed knee and ankle angles, whereas neuromuscular assessments and their reliability during or between experimental sessions may be modified by changes in joint angles [12,18–25]. Indeed, PF MVC decreases with knee flexion or plantar flexion with no change in muscle activation [18,21–24], whereas the PF H_max_/M_max_ appears to be lower when the ankle angle is greater than 90° [19,20,26]. It was also reported that the reliability of some neuromuscular assessments of PF (H_max_, M_max_, and H_max_/M_max_) are modified when changing the position of the ankle joint [12], suggesting that changes in the muscle length could also influence the reliability of neuromuscular assessments. To our knowledge, a comparison of the reliability of PF neuromuscular tests at different knee angles has never been investigated. The soleus (SOL) muscle only crosses ankle joint (mono-articular), whereas the gastrocnemii cross ankle and knee joints (bi-articular), indicating that a change in the knee angle could influence the reliability of PF neuromuscular tests. Therefore, it is relevant to identify the knee angle that provides the best reliability for neuromuscular assessments.

The purpose of this study was to evaluate and compare the reliability of PF neuromuscular tests during (intra-session) and between (inter-session) experimental sessions at three different knee angles: 0° (fully extended leg), 30° and 90°. To this aim, the PF MVC, VAL, H_max_, M_max_ were measured for the three knee angles (i.e., 0°, 30° and 90°) at three time points (one hour interval between the first two time points and one week interval between the first and the third time points).

## Materials and methods

### Participants

The minimum sample size needed for the study was defined prior to the experiment, using the G*Power software 3.1.9.2 (Franz Faul, University of Kiel, Kiel, Germany). For an expected ‘‘medium” effect size (f = 0.25), a significance level of 0.05, a power (1-β) of 0.9, and a correlation among repeated measures of 0.7, a required sample size of 12 subjects was obtained to compare PF neuromuscular measurements through the experimental conditions and sessions. This sample size was in accordance with the recommendation of Hopkins et al. (2000) [16] and with previous neuromuscular reliability studies [12,17,27]. The experiments were performed on twelve healthy young adults (7 males and 5 females, age 22.5±1.2 years, height 172.5±9.7 cm, mass 63.5±9.2 kg, mean±SD) with no history of neurological and/or musculoskeletal disorders. All of the subjects were students and were recruited from the University of Nantes (France). They were volunteered to participate in the experiment and were informed of the nature, aims, risks and discomfort associated with the study before they gave their written consent prior to participation in the investigation. The subjects were not engaged in any strenuous locomotor activity for at least 24 h before the experimental sessions. The protocol of the current investigation was approved by the French National Drugs and Health Administration and by the National Ethics Committee section Nantes Ouest IV (ID: 635/2015) and was in conformity with the Declaration of Helsinki (last modified in 2013).

### Experimental protocol

**Fig 1:**
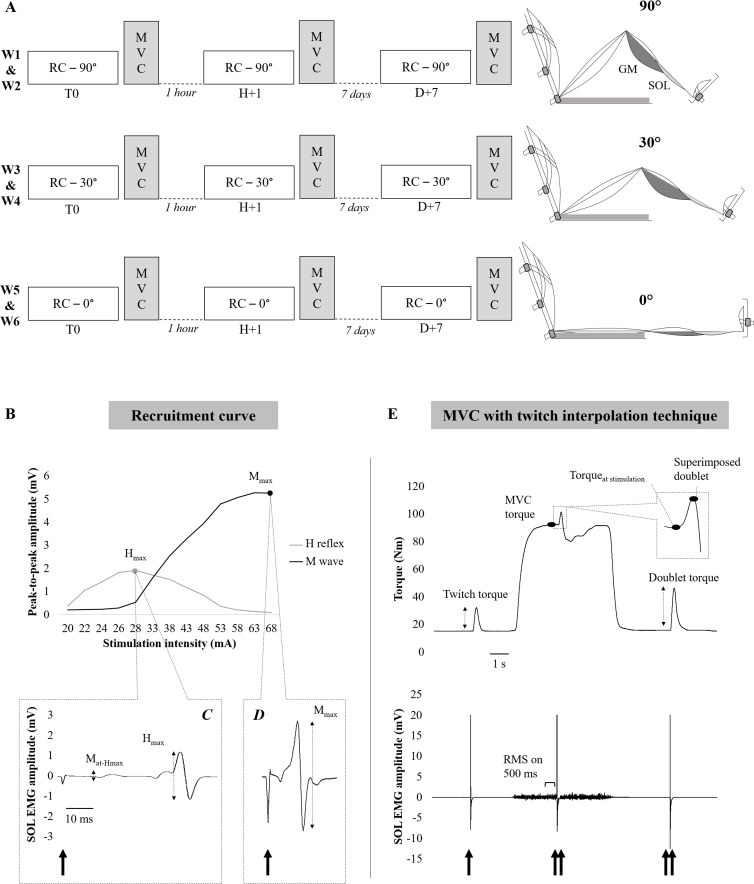
Illustration of the experimental protocol and data analysis. (A) represents the experimental protocol with the three knee angles tested. Each knee angle was tested over two consecutive weeks. (B) represents the recruitment curves of the H reflex (grey line) and M wave (black line). (C) represents a typical SOL EMG trace at a stimulation intensity evoking at the H_max_. (D) represents a typical SOL EMG trace at a stimulation intensity evoking at the M_max_. (E) represents the torque and SOL EMG recordings during the MVC. Single spike represents a single stimulus. Double spikes represent paired stimuli (doublet). MVC: maximal voluntary contraction; W: week; GM: gastrocnemius medialis; SOL: soleus; H_max_: maximal Hoffmann reflex; M_max_: maximal motor potential; M_at-Hmax_: motor potential recorded with the H_max_.

The neuromuscular assessments of PF were carried out for 90°, 30° and 0° knee angles and at three time points for each angle: Time 0 (T0 = first measurement), 1 h after T0 (H+1) and 7 days after T0 (D+7) (Fig 1A); i.e., 6 sessions per subject (2 sessions per angle). The tested angles were randomly administered to the subjects at the same time of day over six consecutive weeks. Thus, the sequences were performed in the following order by one (90°-0°-30°), two (0°-90°-30°, 30°-0°-90°, 30°-90°-0° and 90°-30°-0°) and three participants (0°-30°-90°). For each angle, the subjects were invited to participate in two experimental sessions: the first with tests at T0 and H+1 and the second with tests at D+7. The duration of each session was approximatively 45 min (excluding the subjects’ preparation and installation). Approximatively 15 min rest period where the participant sat comfortably in a chair was therefore respected between the first (T0) and the second session (H+1). A recruitment curve of the H reflex and M wave (Fig 1B) was performed to obtain the H_max_ and M_max_ (Fig 1C and 1D). After the recruitment curve was completed, the subjects performed a standardized warm-up. Then, the subjects performed two PF MVCs separated with a 3 min rest period. Throughout the subjects’ attempts to produce maximal effort, standardized verbal encouragements were given during execution.

Paired stimuli were delivered during (superimposed doublet) and 3 s after the MVC (potentiated doublet) to investigate PF muscle contractile properties and to assess the VAL using the twitch interpolation technique [9] (Fig 1E).

### Data recordings

#### Mechanical recordings

The participants were tested in the seated position with the trunk inclined backward at 20° (0° = vertical). A goniometer was used to set the ankle angle at 90° and the knee angle at 90°, 30° or 0°. Their right foot was secured by two straps to the footplate of a dynamometer (Biodex 3 Pro, Shirley, NY, USA). The centre of rotation of the dynamometer shaft was aligned with the anatomical ankle flexion-extension axis. The subjects were securely stabilized by two crossover shoulder harnesses and a belt across the abdomen. Particular care was taken to monitor the subjects’ posture. They had to keep their hands folded on their chest. They were also asked to avoid head rotations during the test in order to maintain constant cortico-vestibular influences on the excitability of the motor pool and to limit afferent feedback from other peripheral receptors, i.e., Golgi tendon organs, or cutaneous and joint afferents [28,29].

#### Electromyography recordings

The subjects’ skin was first carefully prepared by shaving, abrading and cleaning with alcohol. Then, bipolar silver chloride (Ag-AgCL) surface electrodes (Kendall Medi-Trace™, Canada) of 1-mm diameter with an inter-electrode distance (centre-to-centre) of 2 cm were placed along the mid-dorsal line of the right leg, ~5 cm below the insertion of the two heads of the gastrocnemii on the Achilles tendon for the SOL measurements. Gastrocnemius medialis (GM) recording electrodes were fixed lengthwise over the middle of the muscle belly. The GM was chosen rather than the gastrocnemius lateralis because its behaviour through experimental conditions was reported to be similar to the gastrocnemius lateralis [3,30,31] and neuromuscular measurements are more reliable on this muscle [17]. The reference electrodes were placed on the patella of the left leg. The placement of the electrodes was marked on the skin with an indelible pen to ensure that the same recording site was used in the subsequent experimental sessions (i.e., during the six weeks). The EMG signal was amplified using a bandwidth frequency ranging from 5 Hz to 1 kHz (gain = 500), sampled at 2 kHz using the Biopac acquisition system (MP35, BIOPAC, Goleta, USA) and stored with commercially available software (BIOPAC student Lab Pro, Biopac Systems Inc., Goleta, USA) for off-line analysis.

#### Electrical stimulation

The SOL and GM electrophysiological responses, the H reflex and M wave, were evoked by percutaneous stimulation of the posterior tibial nerve with a single rectangular pulse (1 ms) and a high voltage (400 V), which was automatically delivered by a Digitimer stimulator (Model DS7-AH, Hertfordshire, UK). The self-adhesive cathode (1 cm diameter, Ag-AgCl) was placed in the popliteal fossa and the anode (5 x 10 cm, Medicompex SA, Ecublens, Switzerland) on the anterior surface of the knee. At the beginning of each experimental session, the optimum cathode position, namely the site where the greatest H reflex amplitude in the SOL was evoked for a stimulation intensity of 30 mA, was located with a hand-held cathode ball (0.5 cm diameter). Once the position was determined, the cathode electrode was fixed to this site using tape for reducing the pain experienced during electrical stimulation [32]. The recordings of the H reflex and M wave recruitment curves were then started from the SOL H reflex threshold. The stimulation intensity was increased in 2 mA increments until the M_max_ was obtained (Fig 1B, 1C and 1D). Four stimuli were delivered at each intensity because this number of stimuli is optimal to obtain high H reflex reliability [33,34]. Stimuli were interspaced by a 10 s interval to avoid the confounding effect of homosynaptic post-activation depression [35]. The maximal intensity of the stimulation (i.e., intensity which recruited all PF motor units) was reached when the amplitude of the twitch force and the peak-to-peak amplitude of the SOL and GM M wave plateaued. Once the optimal intensity was found, 120% of this intensity was used in the application of single and paired stimuli during and after the MVC efforts.

### Data analysis

#### H reflex and M wave recruitment curves

For the SOL and GM, the peak-to-peak amplitude of the H_max_, the M_at-Hmax_ (i.e., the M wave recorded with the H_max_) and the M_max_ (Fig 1C and 1D) were calculated as the mean over the four recordings in each experimental condition. The H_max_/M_max_ was calculated to assess the proportion of motor units that were activated by the Ia afferents and potential changes in the balance between excitation and inhibition at the spinal level [29,36–38]. To control that the same proportion of α-motoneurons was activated by the electrical stimulation in each experimental session, the M_at-Hmax_/M_max_ ratio was compared [3]. Twitch peak torque was measured from the twitch associated with the M_max_ stimulation (4 recordings for each recruitment curve). Then, a mean of the 4 twitch peak torques was considered for further analysis.

#### Maximal voluntary contraction

The MVC was considered as the highest peak torque value measured over two trials. The potentiated doublet peak torque was evoked using electrical paired stimuli 3 s after the end of the MVC (Fig 1E) [39]. The maximal VAL was quantified by measuring the superimposed torque response to nerve stimulation during the MVC effort [40,41]. The VAL was estimated according to the following formula, including the Strojnik and Komi [42] correction:

$$VAL=\left(1-\frac{superimposed\;doublet\;x\;\frac{{Torque}_{at\;stimulation}}{MVC\;torque}}{Potentiated\;doublet}\right)x100$$

The maximal EMG (EMG_max_) for the MVC of the SOL and GM was quantified as the root mean square value over a 0.5 s interval around the peak MVC torque (Fig 1E). The EMG_max_ values were then normalized to the respective M wave amplitude for the respective muscles to obtain the EMG_max_/M_max_ ratio [2].

### Statistical analysis

The statistical analyses were performed using Statistica 10 (Statsoft, Tulsa, OK, USA). A significance level of *P*<0.05 was used for all analyses. All data are presented as the means ± standard deviation (SD). Three-factor ANOVAs with repeated measures [time (T0, H+1 and D+7) x muscle (SOL and GM) x knee angle (90°, 30° and 0°)] were performed for the H_max_, M_max_, H_max_/M_max_, M_at-Hmax_/M_max_, and EMG_max_/M_max_. Two-factor ANOVAs [time (T0, H+1 and D+7) x knee angle (90°, 30° and 0°)] were performed for the MVC torque, potentiated doublet torque, twitch torque and VAL. The effect size for each ANOVA was also calculated as partial eta square ($\eta_{p}^{2}$). When a main effect or a significant interaction was found, a post-hoc analysis was made using Tukey’s test. Relative reliability is the degree to which individuals maintain their position in a sample with repeated measurements; and absolute reliability is the degree to which repeated measurements vary for individuals [43]. Indices of relative reliability, the intraclass correlation coefficient (ICC), and absolute reliability, the coefficient of variation (CV) and the standard error of measurement (SEM), were calculated to assess the intra-session (T0 vs. H+1 measurements) and inter-session (T0 vs. D+7 measurements) reliability of the neuromuscular assessment-related variables. The ICC (3,1) was chosen from Shrout and Fleiss (1979) [44,45]. We denoted ICC values < 0.4 as poor, 0.4–0.59 as fair, 0.60–0.74 as good, and > 0.75 as excellent [46–48]. The CV was defined as (*s*/mean) ∙ 100, where *s* is the standard deviation and mean is the mean of the change scores of the measure [43]. The SEM was calculated as the SD of the differences between the two measurements divided by the square root of the number of measurements ($\mathrm{SD}/\sqrt{2}$).

## Results

### Effect of the knee angle on the neuromuscular assessment

The electrophysiological and mechanical data are presented in Tables 1 and 2, respectively.

**Table 1:** Intra- and inter-session reliability of electrophysiological measurements through three knee angles.

		90° of knee angle				30° of knee angle				0° of knee angle			
		***Intra-session (T0 vs H+1)***											
		**Mean (SD)**	**ICC**	**CV (SD)**	**SEM**	**Mean (SD)**	**ICC**	**CV (SD)**	**SEM**	**Mean (SD)**	**ICC**	**CV (SD)**	**SEM**
***SOL***	**H**_**max**_ **(mV)**	5.8 (3.0) vs 7.1 (3.8)	0.84	16.3 (15.0)	1.49	6.0 (2.9) vs 7.4 (4.0)	0.80	20.3 (20.5)	1.66	6.2 (4.2) vs 6.9 (5.1)	0.89	19.1 (17.7)	1.75
	**M**_**max**_ **(mV)**	12.4 (3.2) vs 13.7 (3.7)	0.98	6.9 (3.7)	0.54	13.2 (4.8) vs 14.3 (4.8)	0.98	7.2 (8.0)	0.82	13.4 (6.3) vs 14.2 (6.4)	0.99	6.5 (6.8)	0.73
	**H**_**max**_**/M**_**max**_ **(%)**	47.4 (23.4) vs 51.5 (23.6)	0.91	12.2 (11.5)	7.83	47.0 (22.8) vs 50.5 (21.0)	0.77	18.4 (17.4)	11.31	44.9 (18.3) vs 48.8 (24.9)	0.87	16.6 (16.2)	8.69
	**M**_**at-Hmax**_**/M**_**max**_ **(%)**	12.6 (9.0) vs 17.9 (17.7)	0.46	48.4 (34.5)	10.72	11.8 (9.9) vs 11.8 (7.3)	0.15	26.4 (29.1)	8.05	7.9 (6.4) vs 11.8 (7.3)	0.34	51.9 (30.3)	5.68
	**EMG**_**max**_**/M**_**max**_	3.1 (1.4) vs 3.5 (1.4)	0.69	15.7 (16.4)	0.85	2.9 (1.1) vs 3.6 (1.5)	0.61	24.7 (10.3)	0.88	3.4 (1.6) vs 2.8 (1.3)	0.85	15.8 (14.9)	0.61
		***Inter-session (T0 vs D+7)***											
	**H**_**max**_ **(mV)**	5.8 (3.0) vs 5.4 (2.8)	0.91	15.6 (7.4)	0.98	6.0 (2.9) vs 5.6 (4.5)	0.57	30.8 (27.2)	2.61	6.2 (4.2) vs 6.2 (3.7)	0.87	21.0 (13.5)	1.53
	**M**_**max**_ **(mV)**	12.4 (3.2) vs 11.6 (4.3)	0.84	13.7 (8.8)	1.64	13.2 (4.8) vs 12.0 (3.8)	0.89	12.0 (7.3)	1.57	13.4 (6.3) vs 13.6 (5.0)	0.94	9.79 (6.93)	1.57
	**H**_**max**_**/M**_**max**_ **(%)**	47.4 (23.4) vs 47.9 (23.6)	0.94	11.0 (8.8)	6.36	47.0 (22.8) vs 45.1 (27.9)	0.71	27.3 (27.4)	14.68	44.9 (18.3) vs 44.7 (20.2)	0.88	15.7 (14.6)	7.45
	**M**_**at-Hmax**_**/M**_**max**_ **(%)**	12.6 (9.0) vs 13.8 (12.0)	0.91	22.0 (12.3)	3.51	11.8 (9.9) vs 17.5 (10.4)	0.32	41.1 (30.6)	8.53	7.9 (6.4) vs 13.8 (13.1)	0.45	46.6 (33.9)	7.87
	**EMG**_**max**_**/M**_**max**_	3.1 (1.4) vs 3.2 (1.5)	0.53	19.6 (20.1)	1.04	2.9 (1.1) vs 3.0 (1.4)	0.62	21.3 (11.9)	0.82	3.4 (1.6) vs 3.8 (2.7)	0.51	25.0 (16.8)	1.62
		***Intra-session (T0 vs H+1)***											
***GM***	**H**_**max**_ **(mV)**	2.2 (1.5) vs 2.6 (1.6)	0.94	17.1 (14.2)	0.43	1.5 (0.8) vs 1.6 (0.7)	0.63	21.2 (15.9)	0.46	1.4 (0.9) vs 1.4 (1.0)	0.88	24.5 (15.6)	0.38
	**M**_**max**_ **(mV)**	7.4 (3.5) vs 7.7 (3.6)	0.94	10.6 (10.6)	0.95	7.5 (3.2) vs 7.7 (2.9)	0.91	9.9 (8.9)	1.02	10.7 (3.9) vs 9.9 (3.7)	0.95	9.3 (11.6)	0.98
	**H**_**max**_**/M**_**max**_ **(%)**	31.7 (23.0) vs 35.7 (22.2)	0.94	15.0 (12.7)	5.95	23.4 (17.1) vs 22.8 (13.2)	0.81	20.6 (12.9)	7.24	13.0 (7.2) vs 13.7 (7.1)	0.67	26.9 (19.6)	4.32
	**M**_**at-Hmax**_**/M**_**max**_ **(%)**	69.0 (38.7) vs 58.9 (38.6)	0.86	32.0 (42.1)	15.73	59.9 (40.6) vs 55.8 (39.6)	0.95	20.9 (18.4)	10.37	44.7 (30.8) vs 58.8 (36.2)	0.52	38 (41.5)	24.23
	**EMG**_**max**_**/M**_**max**_	2.5 (1.4) vs 3.6 (2.6)	0.47	27.9 (27.9)	1.59	4.9 (3.0) vs 4.2 (1.7)	0.73	16.7 (14.3)	1.34	4.0 (1.8) vs 3.6 (1.9)	0.98	10.4 (6)	0.32
		***Inter-session (T0 vs D+7)***											
	**H**_**max**_ **(mV)**	2.2 (1.5) vs 2.0 (1.1)	0.90	16.2 (10.4)	0.48	1.5 (0.8) vs 1.3 (0.7)	0.90	19.7 (23.9)	0.25	1.4 (0.9) vs 1.3 (0.7)	0.40	34.9 (19.5)	0.66
	**M**_**max**_ **(mV)**	7.4 (3.5) vs 7.8 (4.5)	0.87	15.4 (12.4)	1.56	7.5 (3.2) vs 8.0 (3.4)	0.90	12.2 (8.0)	1.16	10.7 (3.9) vs 11.0 (4.1)	0.94	8.0 (6.7)	1.08
	**H**_**max**_**/M**_**max**_ **(%)**	31.7 (22.9) vs 29.3 (17.7)	0.82	20.2 (11.7)	9.53	23.4 (17.1) vs 18.6 (14.6)	0.93	28.9 (24.2)	4.68	13.0 (7.2) vs 12.7 (7.4)	0.33	35.9 (21.1)	6.08
	**M**_**at-Hmax**_**/M**_**max**_ **(%)**	69.0 (38.7) vs 60.3 (35.5)	0.45	45.6 (43.2)	28.4	59.9 (40.6) vs 66.0 (35.6)	0.23	50.5 (39)	34.06	44.7 (30.8) vs 46.7 (32.4)	0.40	61.2 (47.3)	25.25
	**EMG**_**max**_**/M**_**max**_	2.5 (1.4) vs 2.8 (1.5)	0.43	27.3 (22.9)	1.12	4.9 (3.0) vs 4.1 (2.2)	0.91	16.2 (8.8)	0.87	4.0 (1.8) vs 4.0 (1.5)	0.02	23.4 (22.2)	1.65

SOL: soleus; GM: gastrocnemius medialis; H_max_: maximal Hoffmann reflex; M_max_: maximal motor potential; M_at-Hmax_: motor potential recorded with the H_max_; EMG_max_: EMG activity during the MVC; SD: standard deviation; ICC: interclass correlation coefficient; CV: coefficient of variation; SEM: standard error of measurement.

**Table 2:** Intra- and inter-session reliability of mechanical measurements through three knee angles.

	90° of knee angle				30° of knee angle				0° of knee angle			
	***Intra-session (T0 vs H+1)***											
	**Mean (SD)**	**ICC**	**CV (SD)**	**SEM**	**Mean (SD)**	**ICC**	**CV (SD)**	**SEM**	**Mean (SD)**	**ICC**	**CV (SD)**	**SEM**
**MVC torque (N.m)**	85.2 (30.4) vs 92.0 (29.7)	0.97	7.9 (6.4)	6.10	136.4 (37.7) vs 142.4 (42.4)	0.93	5.9 (5.6)	11.97	140.7 (45.4) vs 142.1 (43.6)	0.96	7.2 (5.4)	10.22
**Twitch torque (N.m)**	15.4 (4.4) vs 15.5 (4.6)	0.98	3.7 (2.0)	0.79	20.8 (4.5) vs 20.7 (5.4)	0.92	5.3 (4.0)	1.59	22.8 (5.6) vs 23.0 (5.9)	0.96	4.0 (3.4)	1.23
**Doublet torque (N.m)**	27.4 (7.1) vs 27.5 (7.7)	0.94	5.5 (4.0)	1.99	37.5 (8.8) vs 36.5 (10.1)	0.98	3.9 (3.5)	1.52	40.3 (8.6) vs 40.5 (8.8)	0.97	2.7 (2.8)	1.63
**VAL (%)**	82.5 (12.2) vs 85.2 (11.4)	0.83	4.4 (3.5)	5.29	88.8 (12.1) vs 92.1 (9.7)	0.21	7.9 (11.6)	9.82	90.0 (13.9) vs 90.1 (11.3)	0.84	6.0 (5.32)	5.63
	***Inter-session (T0 vs D+7)***											
**MVC torque (N.m)**	85.2 (30.4) vs 88.9 (34.6)	0.88	11.8 (10.2)	12.23	136.4 (37.7) vs 134.4 (42.0)	0.82	10.9 (11.7)	18.65	140.7 (45.4) vs 151.6 (57.6)	0.77	16.7 (11.5)	26.69
**Twitch torque (N.m)**	15.4 (4.4) vs 14.8 (3.6)	0.90	6.5 (6.4)	1.40	20.8 (4.5) vs 20.4 (4.5)	0.80	8.2 (5.3)	2.17	22.8 (5.6) vs 22.7 (4.9)	0.91	5.7 (4.4)	1.72
**Doublet torque (N.m)**	27.4 (7.1) vs 27.2 (7.2)	0.87	7.3 (6.3)	2.81	37.5 (8.8) vs 37.7 (8.0)	0.93	4.9 (3.5)	2.47	40.3 (8.6) vs 42.2 (10.2)	0.85	7.9 (5.6)	3.95
**VAL (%)**	82.5 (12.7) vs 82.9 (14.9)	0.38	9.6 (10.8)	11.02	88.8 (12.1) vs 84.9 (18.2)	0.74	8.2 (10.5)	8.43	90.0 (13.9) vs 89.4 (13.9)	0.35	6.0 (6.7)	11.43

MVC: maximal voluntary contraction; VAL: voluntary activation level; SD: standard deviation; ICC: interclass correlation coefficient; CV: coefficient of variation; SEM: standard error of measurement.

#### Electrophysiological measurements

A significant interaction between muscle and time was found for the H_max_ (*F*_*(2*,*22)*_ = 5.7; *P*<0.01; $\eta_{p}^{2}$ = 0.34); however, no angle effect (*F*_*(2*,*22)*_ = 0.4; *P* = 0.70; $\eta_{p}^{2}$ = 0.03) was observed. The SOL H_max_ was significantly (*P*<0.01) higher at H+1 compared with T0 and D+7, whereas no difference was observed for the GM H_max_. The SOL H_max_ was significantly (*P*<0.001) higher than the GM H_max_ at each of the three time points of measurement.

A significant effect of angle (*F*_*(2*,*22)*_ = 6.4; *P*<0.01; $\eta_{p}^{2}$ = 0.37) and an interaction between muscle and time were found for the M_max_ (*F*_*(2*,*22)*_ = 18.7; *P*<0.001; $\eta_{p}^{2}$ = 0.63). The M_max_ was significantly (*P*<0.01) lower for the 90° and 30° knee angle compared with 0°. The SOL M_max_ was significantly (*P*<0.01) higher at H+1 compared with T0 and D+7, whereas no difference was observed for the GM M_max_. The SOL M_max_ was significantly (*P*<0.01) higher than the GM M_max_ at each of the three time points of measurement.

**Fig 2:**
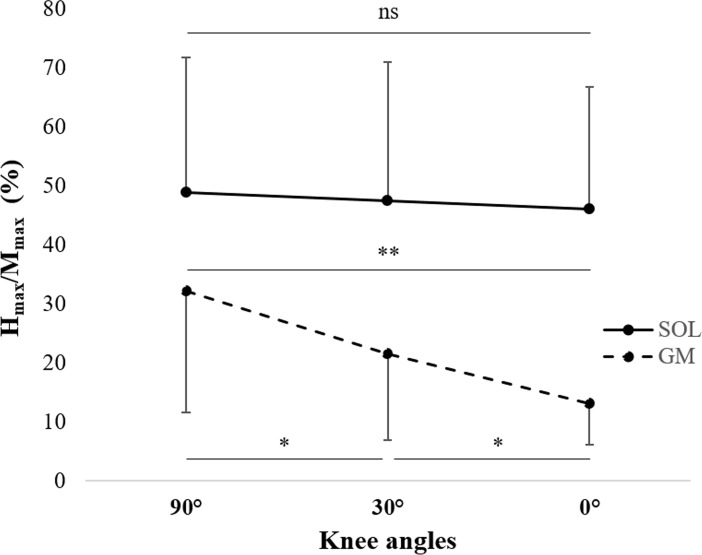
Effect of the knee angle on the soleus (SOL, *continuous lines)* and gastrocnemius medialis (GM, *dotted lines)* H_max_/M_max_. ns: no statistical difference. * *P*<0.05, ** *P*<0.01.

A significant interaction between muscle and angle was found for the H_max_/M_max_ (*F*_*(2*,*22)*_ = 10.8; *P*<0.001; $\eta_{p}^{2}$ = 0.50). No change in the H_max_/M_max_ was observed for the SOL. For the GM it was significantly (*P*<0.05) higher for the 90° knee angle compared with 30° and 0°, while it was higher for 30° than 0° (Fig 2). Only a main effect of muscle (*F*_*(1*,*11)*_ = 49.8; *P*<0.001; $\eta_{p}^{2}$ = 0.82) was found for the M_at-Hmax_/M_max_, revealing that the SOL M_at-Hmax_/M_max_ (13.2 ± 10.8%) was significantly (*P*<0.001) lower than the GM M_at-Hmax_/M_max_ (57.8 ± 36.0%).

A significant muscle x knee angle interaction (*F*_*(2*,*22)*_ = 10.8; *P*<0.05; $\eta_{p}^{2}$ = 0.20) was found for the EMG_max_/M_max_. Although no effect of the knee angle was observed for the SOL, the EMG_max_/M_max_ of the GM was significantly (*P*<0.01) lower for the 90° knee angle compared with 30° and 0°.

#### Mechanical measurements

Only a main effect of angle was found for the MVC torque (*F*_*(2*,*22)*_ = 26.0; *P*<0.001; $\eta_{p}^{2}$ = 0.70), twitch torque (*F*_*(2*,*22)*_ = 54.0; *P*<0.001; $\eta_{p}^{2}$ = 0.83) and the potentiated doublet torque (*F*_*(2*,*22)*_ = 54.1; *P*<0.001; $\eta_{p}^{2}$ = 0.83). The MVC torque for the 90° knee angle was significantly (*P*<0.001) lower than that for 30° and 0°. The twitch torque and potentiated doublet torque were significantly (*P*<0.05) lower for the 90° knee angle compared with 30° and 0°, and lower for 30° than that for 0°. No significant difference was observed for the VAL through the three knee angles and time points.

### Reliability of neuromuscular assessment

The reliability of electrophysiological and mechanical data is presented in Tables 1 and 2, respectively.

#### Electrophysiological measurements

The intra- and inter-session reliability of the M_max_ was excellent regardless of the muscle and the knee angle considered. The intra-session reliability of the SOL and the GM H_max_ was good-to-excellent for the three knee angles. The inter-session reliability was better for the 0° and 90° knee angles compared with 30° for the SOL. For the GM, the inter-session reliability was excellent for the 90° and 30° knee angle, but poor for 0°.

The intra-session reliability of the SOL H_max_/M_max_ was good-to-excellent for the three knee angles. The inter-session reliability of the SOL H_max_/M_max_ was excellent for the 90° and 0° knee angles and only good for the 30° knee angle.

The intra-session reliability of the GM H_max_/M_max_ was good-to-excellent with a high CV for the three knee angles. The inter-session reliability of the GM H_max_/M_max_ was excellent for the 90° and 30° knee angles, while the CV were high, and poor for the 0° knee angle.

The intra-session reliability of the SOL and GM H_max_/M_max_ was good-to-excellent for the three knee angles. The inter-session reliability of the SOL and GM was good-to-excellent for all the knee angles, except at the 0° knee angle for the GM where the reliability was poor.

The intra-session reliability of the SOL M_at-Hmax_/M_max_ was fair or poor for all the knee angles. The inter-session reliability of the SOL M_at-Hmax_/M_max_ was better for the 90° knee angle compared with 30° and 0°. The intra-session reliability of the GM M_at-Hmax_/M_max_ was better for the 90° and 30° knee angles compared with 0°. The inter-session reliability ot the GM M_at-Hmax_/M_max_ was fair or poor for all the knee angles.

The intra-session reliability of the SOL EMG_max_/M_max_ was good-to-excellent for all the knee angles, but the CV were relatively high. The inter-session reliability was fair for the 90° and 0° knee angles and good for the 30° knee angle, but the CV was high. For the GM, the intra-session reliability of the EMG_max_/M_max_ was worst for the 90° knee angle compared with 30° and 0°. The inter-session reliability was better for the 30° knee angle than that for 90° and 0°.

#### Mechanical measurements

The intra- and inter-session reliability of the MVC torque, twitch torque and potentiated doublet torque was excellent regardless of the knee angle considered.

The intra-session reliability of the VAL was better for the 90° and 0° knee angles compared with 30°. The inter-session reliability of the VAL reliability was better for the 30° knee angle than that for 0° and 90°. However, for the intra- and inter-session reliability, all the CV of the VAL were low regardless of the knee angle considered, which showed low intra-subject variability.

## Discussion

This study was the first to examine the effect of knee angle on the reliability of the PF neuromuscular assessment. The main findings of this investigation were that: i) the M_max_, H_max_/M_max_, MVC, twitch and doublet torque were the most reliable measurements; and ii) the reliability of electrophysiological measurements seems to be better for the 90° knee angle than that for 0° and 30°.

### Effect of the knee angle on the neuromuscular assessments

The present study demonstrated that the knee angle influences mechanical and electrophysiological measurements of PF muscles. A decrease in the PF MVC torque was observed for the 90° knee angle compared to 0°. These results are in agreement with previous findings showing a decrease in force generated by PF muscles with knee flexion [18,49]. The similar decrease in PF twitch torque and potentiated doublet torque with decreasing knee angle was found. This confirmed, as suggested by Cresswell et al. (1995) [18], that the decrease in the MVC torque observed with decreasing knee angle was, in part, a result of changes in the contractile behaviour of the GM and/or neuromuscular transmission-propagation. Based on the crossed-bridge theory [50], the GM sarcomere length would be more optimal at 90° knee angle than at 0° to enable cross-bridge interactions between myosin and actin filaments. The PF VAL was not affected by changes in the knee angle, which suggests that the ability of the central nervous system to recruit a maximum of motor units remains constant regardless of the knee angle considered [24]. The present results reported however, in agreement with previous observations [51], an altered neural drive (EMG_max_/M_max_) of the GM when the leg was the most flexed (i.e. 90° vs 30° and 0° knee angle). This result indicated therefore that a reduced neural drive for the gastrocnemii can be in part involved in the decrease in the maximal force production of the PF muscles with knee flexion.

Concerning the electrophysiological responses, the results showed a lack of change in M_at-Hmax_/M_max_ revealing that the same proportion of α-motoneurons was activated by the electrical stimulus in each condition [3,28,52]. It can be therefore considered that the H_max_/M_max_ of each PF muscles were comparable though the three knee angles. GM H_max_/M_max_ were lower at the 0° (extended leg) knee angle compared to 90° (flexed leg). Leg extension increases the muscle length of the GM [20,53], which facilitates presynaptic inhibition and leads to reduced efficacy of the spinal transmission from Ia afferents to α-motoneurons [19,54,55]. In contrast, no variation was observed for the SOL H_max_/M_max_ with knee extension. This finding was different compared to the work of Ushiyama et al. (2010) [20] in which a decrease in the SOL H_max_/M_max_ with increasing knee extension was observed. In agreement with previous findings [18], a lower GM M_max_ was observed at 90° compared to 0°. Cresswell et al. (1995) [18] suggested that, in the shortened position, the diameter of each muscle fibre increases, which would reduce the number of muscle fibres in the recording volume of the electrodes.

### Angle effect on the reliability of the electrophysiological assessments

Stutzig and Siebert (2016) already investigated the reliability of PF electrophysiological measurements over a period of 2 weeks [17]. They observed, at a fixed knee (100°) and ankle (90°) angle, that SOL measurements were more reproducible than the GM measurements and conclude, nevertheless, that SOL and GM may be selected for longitudinal studies of the adaptations of the triceps surae. Chen et al. (2010) showed that the reliability of SOL electrophysiological measurements was affected by changes in ankle angle [12], suggesting that muscle length may influence the reproducibility. Because changes in knee angle affect muscle length for the GM but not for the SOL, we have proposed to assess the reliability of GM and SOL electrophysiological measurements through various knee angles. In addition, Chen et al. (2010) [12] and Stutzig and Siebert (2016) [17] did not evaluate the intra-session reliability of their measurements that would provide outcomes about the variability of neuromuscular measurements within an experimental session. Therefore, this study also aimed to assess the intra-session reliability of PF neuromuscular measurements. The results showed that the intra- and inter-session reliability of the M_max_ was excellent regardless of the muscle and knee angle considered. Previous investigations have reported excellent reliability of the SOL M_max_ [12,13,17,56] and GM M_max_ [17] regardless of the knee position considered.

It is well known that the H reflex is a neurological response that has high variability [57]. In line with these findings, our results showed that the H_max_ was less reliable than the M_max_. Although the intra- and inter-session ICC were relatively satisfactory for the H_max_ SOL and GM, it should be noted that the CV were particularly high (i.e., from 15.6% to 34.9%), which indicated an important variability of H reflex measurements regardless of the knee angle considered.

Furthermore, the SOL H_max_ and M_max_ were significantly higher at H+1 compared with T0 and D+7, whereas no difference was observed for the GM H_max_ and M_max_. These measures are likely to vary significantly over the period of an experiment [58–60]. However, when the H_max_ was normalized to the corresponding M_max_ (i.e., H_max_/M_max_), no significant difference between the sessions were found [58].

The intra- and inter-session ICC of the H_max_/M_max_ was good-to-excellent for both muscles, except for the inter-session ICC of the GM muscle for the 30° knee angle. However, the CV was relatively high for the SOL (i.e., from 11.0% to 27.3%), and for the GM (i.e., from 25.0 to 35.9%). The high variability of the H_max_/M_max_ during or between experimental sessions appears to be the result of greater variability of the H_max_, as mentioned above. Moreover, our results reported changes in reliability of the GM H_max_/M_max_ related to the knee angle considered. Excellent reliability of the GM H_max_/M_max_ was observed for the 90° and 30° knee angle compared to 0° (good intra-session, poor inter-session). Chen et al. (2010) [12] also observed a change in the reliability of the H reflex with respect to the ankle angle considered for the SOL. The authors reported higher reliability of the SOL H_max_/M_max_ when the PF muscles were in the shortened position (ICC = 0.96) than when they were stretched (ICC = 0.75). Although the mechanisms responsible for this change in H_max_/M_max_ reliability due to the muscle length is not known, a few authors have observed an increase in inhibitory pathways when muscles are stretched [54,61]. This may imply greater variability in synaptic transmission, which could reduce the reliability. Our results are consistent with the hypothesis proposed by Chen et al. (2010) [12] for the GM muscle, namely that knee extension is not only associated with an increase in inhibitory pathways (-59% reduction of the H_max_/M_max_ between 90° and 0°, *P*<0.01) but also a decrease in reliability (ICC_GM90°_ = 0.94 *vs*. ICC_GM0°_ = 0.67 in intra-session; ICC_GM90°_ = 0.82 *vs*. ICC_GM0°_ = 0.33 in inter-session). In contrast, the reliability of the SOL H_max_/M_max_ was not modified by changes in the knee angle. The SOL is a mono-articular muscle that only crosses the ankle, whereas the GM crosses both the knee and ankle joints and is pluriarticular. Changes in the knee angle do not modify the biomechanical configuration of the SOL. Therefore, the variability in synaptic transmission for the SOL should remain constant while the knee angle is modified, which explains the stability of the SOL H_max_/M_max_ reliability.

The reliability of the SOL and GM EMG_max_/M_max_ was modified by changes in the knee angle and between time sessions. The intra-session reliability of the SOL and GM EMG_max_/M_max_ was good-to-excellent for all the knee angles, except for the GM at 90° (poor). The inter-session reliability was better for the 30° knee angle (good-to-excellent) than that for 0° (poor-to-fair) and 90° (fair). The variability of these measures is very important (15.7<CV<27.9%), except for the intra-session reliability at 0° for both muscles (10.4<CV<15.8%). Overall, this parameter may be considered relatively unreliable, as previously suggested [62].

### Angle effect on the reliability of the mechanical assessments

The intra- and inter-session reliability of the MVC, twitch and doublet torque was excellent (ICC > 0.77) regardless of the knee angle considered. In addition, the results did not reveal a time effect. Similar results have been found by Chen et al. (2010) [12] with a week apart (MVC, ICC = 0.96 with the ankle at 0°), Stutzig et al. (2016) [17] with two weeks apart (MVC, ICC = 0.92; twitch torque, ICC = 0.79) and Clark et al. (2007) with four weeks apart (MVC, ICC = 0.97; twitch torque, ICC = 0.8; potentiated doublet torque, ICC = 0.79), which suggests high reliability of these measurements over time regardless of the knee angle considered. The results showed that the intra-session reliability of the VAL was better for the 90° (excellent) and 0° (excellent) knee angles compared with 30° (poor). The inter-session reliability of the VAL was better for the 30° knee angle (good) than that for 0° (poor) and 90° (poor). Several authors have shown that the magnitude of the ICC tends to decrease when the between-subjects variability is low in the data [16,45]. Therefore, the observed low levels of variability between the subjects may be implicated in the poor intra-session reliability found for the 30° knee angle and inter-session reliability for the 0° and 90° knee angles. Moreover, the intra-session (4.4<CV<7.9%) and inter-session (6.0<CV<9.6%) variability of the VAL was low regardless of the knee angle considered. These results are consistent with those of Clark et al. (2007) [11], Todd et al. (2004) [10] and Stutzig et al. (2016) [17], who reported low CV values for the PF VAL. Our results demonstrated that the twitch interpolation technique is a reliable method to assess the VAL of PF muscles over a period of one week regardless of the knee angle considered.

### Practical applications

Overall, our results revealed good intra- and inter-session reliability of the mechanical assessments. With the ICC above 0.70 (excellent reliability) for each tested knee angle, the MVC, twitch and potentiated doublet torque (i.e. mechanical data) represented the most reliable parameters. The findings showed that the mechanical parameters of PF could be assessed and compared through an experimental session and between sessions regardless of the knee angle considered. Concerning the electrophysiological assessments, the M_max_ and H_max_/M_max_ were highly reliable. To optimize the neuromuscular assessments of PF muscles in young healthy participants, we recommend placing the participants at a 90° knee angle for longitudinal or interventional studies (follow-up measurements). It should be acknowledged that researchers and clinicians should take in account the results of the present study in their outcomes. Indeed, their results can be explained by biological mechanisms, only when the observed changes are greater than the variation of the measurements, i.e. coefficient of variations, observed in the present study.

## Conclusion

To date, the effect of the knee angle on the reliability of neuromuscular assessments of PF muscles was unknown. Our findings demonstrated that changes in the knee angle can influence the reliability of neuromuscular assessments, which reveals the importance of considering this parameter in future studies to collect reliable outcomes.

## References

[1] Gandevia SC. Spinal and supraspinal factors in human muscle fatigue. Physiol Rev. 2001;81: 1725–89. doi: 10.1152/physrev.2001.81.4.1725 11581501 10.1152/physrev.2001.81.4.1725

[2] Millet GY, Martin V, Martin A, Vergès S. Electrical stimulation for testing neuromuscular function: from sport to pathology. Eur J Appl Physiol. 2011;111: 2489–500. doi: 10.1007/s00421-011-1996-y 21590274 10.1007/s00421-011-1996-y

[3] Cattagni T, Martin A, Scaglioni G. Is spinal excitability of the triceps surae mainly affected by muscle activity or body position? J Neurophysiol. 2014;111: 2525–32. doi: 10.1152/jn.00455.2013 24647434 10.1152/jn.00455.2013

[4] Baudry S, Penzer F, Duchateau J. Input-output characteristics of soleus homonymous Ia afferents and corticospinal pathways during upright standing differ between young and elderly adults. Acta Physiol (Oxf). 2014;210: 667–77. 24433254 10.1111/apha.12233

[5] Baudry S, Duchateau J. Age-related influence of vision and proprioception on Ia presynaptic inhibition in soleus muscle during upright stance. J Physiol. 2012;590: 5541–5554. doi: 10.1113/jphysiol.2012.228932 22946095 10.1113/jphysiol.2012.228932 PMC3515837

[6] Maffiuletti NA, Martin A, Van Hoecke J, Schieppati M. The relative contribution to the plantar-flexor torque of the soleus motor units activated by the H reflex and M response in humans. Neurosci Lett. 2000;288: 127–130. 10876077 10.1016/s0304-3940(00)01212-x

[7] Rozand V, Grosprêtre S, Stapley PJ, Lepers R. Assessment of Neuromuscular Function Using Percutaneous Electrical Nerve Stimulation. J Vis Exp. MyJoVE Corporation; 2015; 10.3791/52974 PMC4692605 26436986

[8] Kent-Braun JA, Le Blanc R. Quantitation of central activation failure during maximal voluntary contractions in humans. Muscle Nerve. 1996;19: 861–9. doi: 10.1002/(SICI)1097-4598(199607)19:7&lt;861::AID-MUS8&gt;3.0.CO;2-7 8965840 10.1002/(SICI)1097-4598(199607)19:7<861::AID-MUS8>3.0.CO;2-7

[9] Merton PA. Voluntary strength and fatigue. J Physiol. 1954;123: 553–564. 13152698 10.1113/jphysiol.1954.sp005070 PMC1366225

[10] Todd G, Taylor JL, Gandevia SC. Reproducible measurement of voluntary activation of human elbow flexors with motor cortical stimulation. J Appl Physiol. 2004;97. 10.1152/japplphysiol.01336.2003 15033969

[11] Clark BC, Cook SB, Ploutz-Snyder LL. Reliability of techniques to assess human neuromuscular function in vivo. J Electromyogr Kinesiol. 2007;17: 90–101. doi: 10.1016/j.jelekin.2005.11.008 16427317 10.1016/j.jelekin.2005.11.008

[12] Chen Y-S, Zhou S, Cartwright C, Crowley Z, Baglin R, Wang F. Test-retest reliability of the soleus H-reflex is affected by joint positions and muscle force levels. J Electromyogr Kinesiol. 2010;20: 980–7. doi: 10.1016/j.jelekin.2009.11.003 20005127 10.1016/j.jelekin.2009.11.003

[13] Palmieri RM, Hoffman MA, Ingersoll CD. Intersession reliability for H-reflex measurements arising from the soleus, peroneal, and tibialis anterior musculature. Int J Neurosci. 2002;112: 841–50. 12424824 10.1080/00207450290025851

[14] Ali A, Sabbahi MA. Test-retest reliability of the soleus H-reflex in three different positions. Electromyogr Clin Neurophysiol. 2001;41: 209–14. 11441638

[15] Bernecke V, Pukenas K, Imbrasiene D, Mickeviciene D, Baranauskiene N, Eimantas N, et al Test-Retest Cross-Reliability of Tests to Assess Neuromuscular Function as a Multidimensional Concept. J Strength Cond Res. 2015;29: 1972–84. doi: 10.1519/JSC.0000000000000841 25635607 10.1519/JSC.0000000000000841

[16] Hopkins JT, Ingersoll CD, Cordova ML, Edwards JE. Intrasession and intersession reliability of the soleus H-reflex in supine and standing positions. Electromyogr Clin Neurophysiol. 2000;40: 89–94. 10746184

[17] Stutzig N, Siebert T. Reproducibility of electromyographic and mechanical parameters of the triceps surae during submaximal and maximal plantar flexions. Muscle Nerve. 2016;53: 464–70. doi: 10.1002/mus.24767 26173034 10.1002/mus.24767

[18] Cresswell AG, Loscher WN, Thorstensson A. Influence of gastrocnemius muscle length on triceps surae torque development and electromyographic activity in man. Exp Brain Res. 1995;105: 283–290. 7498381 10.1007/BF00240964

[19] Patikas DA, Kotzamanidis C, Robertson CT, Koceja DM. The effect of the ankle joint angle in the level of soleus Ia afferent presynaptic inhibition. Electromyogr Clin Neurophysiol. 2004;44: 503–11. 15646008

[20] Ushiyama J, Wakahara T, Masani K, Kouzaki M, Muraoka T, Fukunaga T, et al Passive knee movement-induced modulation of the soleus H-reflex and alteration in the fascicle length of the medial gastrocnemius muscle in humans. J Electromyogr Kinesiol. 2010;20: 513–22. doi: 10.1016/j.jelekin.2009.09.004 19854065 10.1016/j.jelekin.2009.09.004

[21] Billot M, Simoneau EM, Ballay Y, Van Hoecke J, Martin A. How the ankle joint angle alters the antagonist and agonist torques during maximal efforts in dorsi- and plantar flexion. Scand J Med Sci Sport. 2011;21: e273–81. 10.1111/j.1600-0838.2010.01278.x 21392122

[22] Dalton BH, Allen MD, Power GA, Vandervoort AA, Rice CL. The effect of knee joint angle on plantar flexor power in young and old men. Exp Gerontol. 2014;52: 70–6. doi: 10.1016/j.exger.2014.01.011 24462806 10.1016/j.exger.2014.01.011

[23] Maganaris CN, Baltzopoulos V, Sargeant AJ. Differences in human antagonistic ankle dorsiflexor coactivation between legs; can they explain the moment deficit in the weaker plantarflexor leg? Exp Physiol. 1998;83: 843–855. 9782193 10.1113/expphysiol.1998.sp004164

[24] Simoneau E, Martin A, Van Hoecke J. Effects of joint angle and age on ankle dorsi- and plantar-flexor strength. J Electromyogr Kinesiol. 2007;17: 307–316. doi: 10.1016/j.jelekin.2006.04.005 16793286 10.1016/j.jelekin.2006.04.005

[25] Alrowayeh HN, Sabbahi MA. H-reflex amplitude asymmetry is an earlier sign of nerve root involvement than latency in patients with S1 radiculopathy. BMC Res Notes. 2011;4: 102 doi: 10.1186/1756-0500-4-102 21466665 10.1186/1756-0500-4-102 PMC3078869

[26] Alrowayeh HN, Sabbahi MA, Etnyre B. Similarities and differences of the soleus and gastrocnemius H-reflexes during varied body postures, foot positions, and muscle function: multifactor designs for repeated measures. BMC Neurol. 2011;11: 65 doi: 10.1186/1471-2377-11-65 21635748 10.1186/1471-2377-11-65 PMC3146399

[27] Hopkins JT, Wagie NC. Intrasession and intersession reliability of the quadriceps Hoffmann reflex. Electromyogr Clin Neurophysiol. 2003;43: 85–9. 12661132

[28] Schieppati M. The Hoffmann reflex: a means of assessing spinal reflex excitability and its descending control in man. Prog Neurobiol. 1987;28: 345–376. 3588965 10.1016/0301-0082(87)90007-4

[29] Zehr PE. Considerations for use of the Hoffmann reflex in exercise studies. Eur J Appl Physiol. 2002;86: 455–468. doi: 10.1007/s00421-002-0577-5 11944092 10.1007/s00421-002-0577-5

[30] Gondin J, Duclay J, Martin A. Soleus- and gastrocnemii-evoked V-wave responses increase after neuromuscular electrical stimulation training. J Neurophysiol. 2006;95: 3328–3335. doi: 10.1152/jn.01002.2005 16481458 10.1152/jn.01002.2005

[31] Place N, Duclay J, Lepers R, Martin A. Unchanged H-reflex during a sustained isometric submaximal plantar flexion performed with an EMG biofeedback. J Electromyogr Kinesiol. 2009;19: e395–402. doi: 10.1016/j.jelekin.2009.01.001 19216091 10.1016/j.jelekin.2009.01.001

[32] Cattagni T, Merlet AN, Cornu C, Jubeau M. H-reflex and M-wave recordings: Effect of pressure application to the stimulation electrode on the assessment of evoked potentials and subject’s discomfort. Clin Physiol Funct Imaging. 2017; 10.1111/cpf.12431 28444940

[33] Williams LR, Sullivan SJ, Seaborne DE, Morelli M. Reliability of individual differences for H-reflex recordings. Electromyogr Clin Neurophysiol. 1992;32: 43–9. 1541247

[34] Mynark RG. Reliability of the soleus H-reflex from supine to standing in young and elderly. Clin Neurophysiol. 2005;116: 1400–1404. doi: 10.1016/j.clinph.2005.02.001 15978502 10.1016/j.clinph.2005.02.001

[35] Hultborn H, Illert M, Nielsen J, Paul A, Ballegaard M, Wiese H. On the mechanism of the post-activation depression of the H-reflex in human subjects. Exp brain Res. 1996;108: 450–62. 8801125 10.1007/BF00227268

[36] Palmieri RM, Ingersoll CD, Hoffman MA. The hoffmann reflex: methodologic considerations and applications for use in sports medicine and athletic training research. J Athl Train. 2004;39: 268–77. 16558683 PMC522151

[37] Baudry S, Collignon S, Duchateau J. Influence of age and posture on spinal and corticospinal excitability. Exp Gerontol. 2015;69: 62–69. doi: 10.1016/j.exger.2015.06.006 26055449 10.1016/j.exger.2015.06.006

[38] Tucker KJ, Turker KS. Muscle spindle feedback differs between the soleus and gastrocnemius in humans. Somat Mot Res. 2004;21: 189–197. 10.1080/08990220400012489 15763904

[39] Rozand V, Cattagni T, Theurel J, Martin A, Lepers R. Neuromuscular fatigue following isometric contractions with similar torque time integral. Int J Sports Med. 2015;36: 35–40. doi: 10.1055/s-0034-1375614 25285471 10.1055/s-0034-1375614

[40] Allen GM, Gandevia SC, McKenzie DK. Reliability of measurements of muscle strength and voluntary activation using twitch interpolation. Muscle Nerve. 1995;18: 593–600. doi: 10.1002/mus.880180605 7753121 10.1002/mus.880180605

[41] Gandevia SC, Allen GM, Butler JE, Taylor JL. Supraspinal factors in human muscle fatigue: evidence for suboptimal output from the motor cortex. J Physiol. 1996;490 (Pt 2: 529–36. 8821149 10.1113/jphysiol.1996.sp021164 PMC1158689

[42] Strojnik V, Komi P V. Neuromuscular fatigue after maximal stretch-shortening cycle exercise. J Appl Physiol. 1998;84: 344–350. doi: 10.1152/jappl.1998.84.1.344 9451655 10.1152/jappl.1998.84.1.344

[43] Atkinson G, Nevill AM. Statistical methods for assessing measurement error (reliability) in variables relevant to sports medicine. Sports Med. 1998;26: 217–38. 9820922 10.2165/00007256-199826040-00002

[44] Shrout PE, Fleiss JL. Intraclass correlations: uses in assessing rater reliability. Psychol Bull. 1979;86: 420–8. 18839484 10.1037//0033-2909.86.2.420

[45] Weir JP. Quantifying test-retest reliability using the intraclass correlation coefficient and the SEM. J Strength Cond Res. 2005;19: 231–40. doi: 10.1519/15184.1 15705040 10.1519/15184.1

[46] Fleiss JL, Levin B, Paik MC. Wiley: Statistical Methods for Rates and Proportions, 3rd Edition—Joseph L. Fleiss, Bruce Levin, Myunghee Cho Paik. In: John Wiley & Sons 2003.

[47] Temesi J, Ly SN, Millet GY. Reliability of single- and paired-pulse transcranial magnetic stimulation for the assessment of knee extensor muscle function. J Neurol Sci. 2017;375: 442–449. doi: 10.1016/j.jns.2017.02.037 28320184 10.1016/j.jns.2017.02.037

[48] Cicchetti D V, Sparrow SA. Developing criteria for establishing interrater reliability of specific items: applications to assessment of adaptive behavior. Am J Ment Defic. 1981;86: 127–37. 7315877

[49] Kennedy PM, Cresswell AG. The effect of muscle length on motor-unit recruitment during isometric plantar flexion in humans. Exp brain Res. 2001;137: 58–64. 11310172 10.1007/s002210000623

[50] Huxley AF. Muscle structure and theories of contraction. Prog Biophys Biophys Chem. 1957;7: 255–318. 13485191

[51] Arampatzis A, Karamanidis K, Stafilidis S, Morey-Klapsing G, DeMonte G, Brüggemann G-P. Effect of different ankle- and knee-joint positions on gastrocnemius medialis fascicle length and EMG activity during isometric plantar flexion. J Biomech. 2006;39: 1891–1902. doi: 10.1016/j.jbiomech.2005.05.010 15993886 10.1016/j.jbiomech.2005.05.010

[52] Grospretre S, Martin A. H reflex and spinal excitability: methodological considerations. J Neurophysiol. 2012;107: 1649–1654. doi: 10.1152/jn.00611.2011 22190624 10.1152/jn.00611.2011

[53] Herbert RD, Moseley AM, Butler JE, Gandevia SC. Change in length of relaxed muscle fascicles and tendons with knee and ankle movement in humans. J Physiol. 2002;539: 637–45. doi: 10.1113/jphysiol.2001.012756 11882694 10.1113/jphysiol.2001.012756 PMC2290150

[54] Hwang IS. Assessment of soleus motoneuronal excitability using the joint angle dependent H reflex in humans. J Electromyogr Kinesiol. 2002;12: 361–366. 12223168 10.1016/s1050-6411(02)00034-2

[55] Robinson KL, McComas AJ, Belanger AY. Control of soleus motoneuron excitability during muscle stretch in man. J Neurol Neurosurg Psychiatry. 1982;45: 699–704. 6215465 10.1136/jnnp.45.8.699 PMC1083159

[56] Christie A, Lester S, LaPierre D, Gabriel DA. Reliability of a new measure of H-reflex excitability. Clin Neurophysiol. 2004;115: 116–23. 14706478 10.1016/s1388-2457(03)00306-7

[57] Nozaki D, Nakazawa K, Yamamoto Y. Fractal correlation in human H-reflex. Exp brain Res. 1995;105: 402–10. 7498394 10.1007/BF00233040

[58] Crone C, Johnsen LL, Hultborn H, Orsnes GB. Amplitude of the maximum motor response (Mmax) in human muscles typically decreases during the course of an experiment. Exp brain Res. 1999;124: 265–70. 9928849 10.1007/s002210050621

[59] Tucker KJ, Tuncer M, Türker KS. A review of the H-reflex and M-wave in the human triceps surae. Hum Mov Sci. 2005;24: 667–88. d doi: 10.1016/j.humov.2005.09.010 16337293 10.1016/j.humov.2005.09.010

[60] McNulty PA, Shiner CT, Thayaparan GK, Burke D. The stability of M(max) and H (max) amplitude over time. Exp brain Res. 2012;218: 601–7. doi: 10.1007/s00221-012-3053-4 22418783 10.1007/s00221-012-3053-4

[61] Guissard N, Duchateau J. Neural aspects of muscle stretching. Exerc Sport Sci Rev. 2006;34: 154–158. doi: 10.1249/01.jes.0000240023.30373.eb 17031252 10.1249/01.jes.0000240023.30373.eb

[62] Halaki M, Gi K. Normalization of EMG Signals: To Normalize or Not to Normalize and What to Normalize to? Computational Intelligence in Electromyography Analysis—A Perspective on Current Applications and Future Challenges. InTech; 2012.

